# A FastSurfer Database for Age‐Specific Brain Volumes in Healthy Children: A Tool for Quantifying Localized and Global Brain Volume Alterations in Pediatric Patients

**DOI:** 10.1002/brb3.70689

**Published:** 2025-07-20

**Authors:** Ibrahim Zughayyar, Martin Bauer, Christopher Güttler, Ana Luisa de Almeida Marcelino, Fabienne Kühne, Claudia Buss, Christine Heim, Annette Aigner, Anna Tietze, Andrea Dell'Orco

**Affiliations:** ^1^ Institute of Neuroradiology Charité ‐ Universitätsmedizin Berlin, Corporate Member of Freie Universität Berlin and Humboldt‐Universität zu Berlin Berlin Germany; ^2^ Institute of Medical Psychology Charité ‐ Universitätsmedizin Berlin, Corporate Member of Freie Universität Berlin and Humboldt‐Universität zu Berlin Berlin Germany; ^3^ Department of Neurology Charité ‐ Universitätsmedizin Berlin, Corporate Member of Freie Universität Berlin and Humboldt‐Universität zu Berlin, Movement Disorders and Neuromodulation Unit Berlin Germany; ^4^ Department of Neonatology Charité ‐ Universitätsmedizin Berlin, Corporate Member of Freie Universität Berlin and Humboldt‐Universität zu Berlin Berlin Germany; ^5^ German Center of Mental Health Berlin Potsdam Partner Site Berlin Germany; ^6^ NeuroCure Cluster of Excellence Charité ‐ Universitätsmedizin Berlin, Corporate Member of Freie Universität Berlin and Humboldt‐Universität zu Berlin Berlin Germany; ^7^ Institute of Biometry and Clinical Epidemiology Charité ‐ Universitätsmedizin Berlin, Corporate Member of Freie Universität Berlin, Humboldt‐Universität Zu Berlin Berlin Germany; ^8^ Center For Stroke Research Berlin Charité ‐ Universitätsmedizin Berlin, Corporate Member of Freie Universität Berlin, Humboldt‐Universität Zu Berlin Berlin Germany

**Keywords:** automated segmentation, brain volumetry, FastSurfer, FreeSurfer, hemispheric asymmetries, neuroimaging, pediatric MRI

## Abstract

**Purpose:**

MRI‐based whole‐brain manual segmentation methods are considered the gold standard for brain volumetric analysis, but are time‐consuming and prone to human error. Automated segmentation tools like FreeSurfer can identify differences in brain volumes between healthy and non‐healthy individuals. Deep‐learning‐based segmentation tools, such as FastSurfer, offer faster processing times, but further validation is needed, particularly in pediatric cases. This study aims to compare FastSurfer with FreeSurfer in a pediatric cohort and compare the volume estimates with previously published reference values.

**Methods:**

A multicenter cohort of 448 subjects aged 4–18 years from three centers was used to compare FastSurfer with FreeSurfer. Validation metrics, including the Dice Similarity Coefficient (DSC), relative volume differences (RVD), and intraclass correlation coefficient (ICC), were computed. Hemispheric asymmetries were assessed by calculating a hemispheric asymmetry index.

**Findings:**

The segmentation methods demonstrated high agreement, with a mean DSC across subjects and regions of interest of 0.90 (95% CI: 0.79; 0.95), RVD of 0.3% (95% CI: −7.6%; 7.4%), and ICC of 0.87 (95% CI: 0.52; 0.94). After a visual inspection, which led to the exclusion of 12 subjects with segmentation errors, growth charts for relative volume estimates of 15 anatomical brain regions were generated, revealing varying growth patterns across ages. A potential clinical application is illustrated by plotting a patient's data on these growth charts, showing a specific atrophy pattern.

**Conclusion:**

To our knowledge, this is the first study investigating the use of FastSurfer in volumetric analysis of a pediatric population. Our findings suggest that FastSurfer is a reliable segmentation tool for pediatric data and is particularly promising for clinical practice due to its high accuracy despite rapid processing times. The morphometric data, growth charts, and code are publicly accessible.

## Graphical abstract

This study aims to validate FastSurfer for pediatric brain segmentation against the widely used FreeSurfer. The considered metrics showed high agreement. Age‐specific growth charts for 15 brain regions, revealing varied growth patterns, were also generated

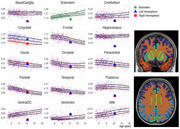

## 1 Introduction

MRI‐based whole‐brain segmentation is the process of partitioning a digital image based on common features of a specific area, representing a fundamental step in detailed quantitative imaging evaluations. Its application in neuroscience includes volumetric measurements, determination of cortical thickness, surface analysis, and the automated detection of brain pathologies. A prominent example is its use for quantifying the hippocampus volume in Alzheimer's disease (Bae et al. 2020; Eskildsen et al. 2012; Ledig et al. 2018; Suh et al. 2020) where it has even been integrated into commercial software solutions (Struyfs et al. 2020) to enhance accessibility in clinical settings.

Volumetric analysis of brain structures is also useful in pediatric cases. In previous studies, it provided valuable insights into genetic disorders (Keppler‐Noreuil et al. 2011; S. Wang et al. 2022), immune‐mediated diseases (Wright et al. 2020), traumatic injuries (Boes et al. 2016; Harkey et al. 2022; Paus et al. 2008; Takayanagi et al. 2013; Z. I. Wang et al. 2016), and cancer treatment (Pancaldi et al. 2023). However, MRI assessments of a child's brain can be particularly challenging, especially for less experienced radiologists, due to the rapid changes in myelination, overall brain, and cerebrospinal fluid (CSF) volumes, and the gray to white matter (GM, WM, respectively) ratio in the early years of life (Phan et al. 2018). As a result, volume loss may be overlooked, or minor asymmetries may be interpreted as atrophy. Objective volumetric measurements are therefore a valuable addition to both clinical practice and research. However, it is important to note that comparisons are only valid with age‐matched peers, highlighting the need for extensive, age‐specific datasets (Bethlehem et al. 2022; Rutherford et al. 2022).

Growth charts are essential for tracking the development of children and adolescents by monitoring height, weight, and head size (Cole 2012). By plotting measurements against standardized growth charts derived from population studies, clinicians could gain insights for detecting deviations indicative of pathologies. This concept can also be applied to individual organs, including the brain. While several large‐scale initiatives track brain MRI data across the lifespan (Bethlehem et al. 2022; Rutherford et al. 2022), they have not used CNN‐based segmentation methods, which offer rapid processing and are ideal for clinical use.

Manual segmentation methods are considered the gold standard for morphometric assessments (Singh and Singh 2021). However, they are tedious, extremely time‐consuming, and prone to human error. Several automated brain segmentation tools have been developed and validated (Fischl et al. 2002; Singh and Singh 2021; Struyfs et al. 2020). Among them, the open‐source software FreeSurfer (Fischl 2012) is particularly well documented and widely used, including for studies involving children and adolescents. Its recon‐all pipeline is a robust processing script that has been applied in numerous studies involving adults (Sulu et al. 2023). FreeSurfer‐based volumetric analysis can also be applied to children and adolescents (Boes et al. 2016; El Marroun et al. 2014; Nickel et al. 2018; Vetter et al. 2020; Yang et al. 2016), provided thorough quality checks are performed (Beelen et al. 2020; Biffen et al. 2020; Ghosh et al. 2010; Pulli et al. 2022; Zivanovic et al. 2021). Although pediatric segmentation pipelines have been developed for research, their adoption in clinical settings has been less widespread, which contrasts with the trend observed in adult populations (J. Y. Lee et al. 2021).

In a clinical setting, it is crucial that patient data can be easily analyzed and that results are promptly available. Therefore, analysis tools must be fast and the results easily interpretable. Deep learning‐based methods, due to their high processing speed, are particularly promising for clinical applications. The FastSurfer pipeline (Henschel et al. 2020; Henschel et al. 2022) is one of these methods, providing cortical and subcortical segmentations in just a few minutes, outperforming the widely used FreeSurfer. While FastSurfer has been validated in adult subjects (ages 18 to 96) from eight publicly available datasets (Henschel et al. 2020), its accuracy and reliability for children and adolescents have yet to be confirmed.

Our study has three main hypotheses: (i) FastSurfer segmentation results in a pediatric population are accurate and precise compared to those obtained using the FreeSurfer pipeline; (ii) FastSurfer‐based growth charts for regional brain volumes reflect the expected volume changes in normally developed children; and (iii) our dataset is representative of brain volumes of children between 4 and 18 years of age. In addition, to demonstrate the clinical relevance of our results, we present an illustrative application of the growth charts. The morphometric data from our cohort, the growth charts, and the accompanying code are made publicly available.

## 2 Material and Methods

### 2.1 Subjects and MRI Data

Three datasets of healthy subjects between the age of 4 and 18 years were used, which included (i) 170 subjects from the publicly available Healthy Brain Network (HBN) (Alexander et al. 2017); (ii) 211 healthy control subjects from the Kids2health study (K2H, kinder‐und‐jugendpsychiatrie.charite.de/forschung/traumafolgen_und_kinderschutz/kids2health/); (iii) 125 subjects from our local MRI (LOC), including patients without morphological alterations in the period between 2015 and 2020. All scans of the LOC group were visually checked by an experienced pediatric neuroradiologist (AT). These subjects had been scanned for the following indications: Uncomplicated headaches, minor head trauma, uncomplicated syncope, dizziness and/or emesis, squint, suspected spinal malformation, and/or bladder voiding dysfunction, suspected malformation in the face or neck region, suspected psychosomatic disorders, and others (anisocoria, somatomegaly, pubertas tarda, suspected myoclonia, muscle weakness, uveitis, tics, drusen, eye movement disorder, family history of aneurysms, memory deficit, small intracranial lipoma, suspected deficit of the visual field, cyst in the pineal region). All scans from the K2H group were visually inspected by a radiologist of the Stroke Research Group of the Charité University Hospital and any neurological pathology was excluded.

The study for the K2H and LOC subjects was approved by the local ethic committees (Charité Universitätsmedizin Berlin EA2/254/20; Ludwig‐Maximilians‐Universität München 18–444). All MRI scans were performed on 3.0 Tesla Siemens MRI systems (HBN on Siemens Tim Trio or Siemens Prisma (Alexander et al. 2017); K2H on Siemens Prisma; LOC on Siemens Skyra). Only the MPRAGE was used for segmentation purposes (selected scan parameters are given in Table 1).

**TABLE 1:** Scan parameters for the local dataset (LOC), Kids2Health (K2H), and HBN dataset (Alexander et al. 2017).

	LOC	K2H	HBN
Voxel size (mm^3^)	0.9 × 0.9 × 0.9	0.8 × 0.8 × 0.8	1.0 × 1.0 × 1.0
TR/TE/TI (ms)	2300/2.3/900	2400/2.2/1000	2500/2.9/1060
Flip angle (^∘^)	8	8	8
Field of view (mm)	240	256	not specified
Acquisition time	5 min 21 s	6 min 38 s	6 min 53s/7 min

### 2.2 MRI Quality Control

DICOM data from LOC were converted to the NIfTI format using dcm2niix (Li et al. 2016), while data from HBN and K2H datasets were received in NIfTI format. The three datasets were merged and organized according to the Brain Image Data Structure (BIDS) guidelines (Gorgolewski et al. 2016).

**FIGURE 1:**
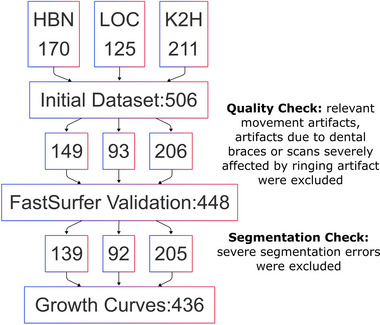
The flow chart illustrates the inclusion and exclusion process of subjects. HBN: Healthy Brain Network; K2H: Kids2health study; LOC: local dataset.

The included datasets were then processed in several steps (Figure 1). Before performing the validation, quality control (QC) was conducted on the MR images by a medical student (IZ), under the supervision of the neuroradiologist (AT). MR images were visually inspected for relevant movement artifacts, artifacts due to dental braces or scans severely affected by ringing artifacts. All images affected by those artifacts were excluded from the validation dataset.

A visual quality assessment of the segmentation was performed to determine unacceptable FastSurfer segmentations. This assessment aimed to ensure that the growth charts were constructed using only accurate segmentations. During this process, the summed masks of 15 regions of interest (ROIs) from both methods were visually inspected to gain insight into the differences between the two methods.

All FastSurfer segmentation results were inspected using FreeSurfer's Freeview. Results showing significant errors, such as major cortical under‐ or over‐segmentations, inaccuracies in the labeling of anatomical regions, or segmentation leakage into vascular structures or CSF spaces were excluded from the final cohort. Minor discrepancies involving only a few voxels were deemed acceptable. It is important to note that for the validation of FastSurfer against FreeSurfer, only the MRIs presenting artifacts were excluded. The inaccurate segmentations were excluded only after the calculation of the validation metrics described below and solely for the construction of the growth charts.

### 2.3 MRI Data Processing

The entire dataset underwent anatomical segmentation using both FreeSurfer v7.4 recon‐all (Fischl 2012) with the hires flag so FreeSurfer conforms to the native resolution and the deep‐learning‐based part of FastSurfer v2.3.3 (Henschel et al. 2022) pipeline. Both analyses were conducted using the original voxel resolution (Table 1). In addition, to compare cortical thickness estimates between the two pipelines, the full FastSurfer pipeline was run in a separate directory. Notably, all volume measurements and growth charts related to FastSurfer refer only to its segmentation component, which does not produce cortical thickness estimates. Exemplary invocations of both pipelines can be found on GitHub (files: kinderseg/mri_processing)

The outputs of both tools include cortical parcellations for 34 bilaterally paired ROIs as defined by the Desikan–Killiany atlas (Desikan et al. 2006), as well as subcortical ROIs. Since such detailed analysis is of limited clinical relevance, we grouped the ROIs into 15 broader anatomical regions (Table S1). Furthermore, the volumes of individual ROIs were normalized by dividing them by the estimated total intracranial volume (eTIV), which encompasses the entire brain volume and CSF spaces (Buckner et al. 2004; Obenaus et al. 2001).

The computational burden of the segmentation processes for both FastSurfer and FreeSurfer was obtained from their respective log files (deep‐seg.log file for FastSurfer, recon‐all.log for FreeSurfer). For FreeSurfer, the duration was calculated by measuring the time difference between the initiation command of recon‐all and the completion of the mri_aparc2aseg command.

For the processing of the data, besides FastSurfer and FreeSurfer, we used Python version 3.9 (Van Rossum and Drake Jr 1995), just as the libraries Pandas v2.0.3 (The Pandas Development Team 2024), NumPy v1.25.2 (Harris et al. 2020), and NiBabel v1.25.2 (Brett et al. 2024). For plots and statistics, we employed R version 4.3.1 (R Core Team 2023), with the packages tidyverse v1.3.0 (Wickham 2016), zoo v1.8.12 (Zeileis and Grothendieck 2005), boot (Davison and Hinkley 1997), and hrbrthemes v0.8.0 (Rudis 2020).

### 2.4 Validation Metrics

To compare the performance of FastSurfer relative to FreeSurfer, we evaluated the segmentation results from both tools using three validation metrics—the dice similarity coefficient (DSC) (Dice 1945; Muller et al. 2022), the relative volume difference (RVD), and the intraclass correlation coefficient (ICC) (Koo and Li 2016).

The DSC measures the overlap between the masks from the two pipelines. Typically, a DSC ≥ 0.8 is considered a very good overlap of the two masks (Kundel and Polansky 2003). The ICC was calculated using a two‐way random effects model [ICC (2,1)]; (Koo and Li 2016). It ranges from 0 to 1, with values close to 1 representing high reliability. An ICC ≥ 0.9 is considered to indicate excellent agreement between measurements, while 0.9> ICC ≥ 0.75 indicates good agreement (Koo and Li 2016). The RVD is calculated for an individual ROI by subtracting the volume estimate derived with FreeSurfer ($V_{\mathrm{FreeSurfer}}$) from the estimate of FastSurfer ($V_{\mathrm{FastSurfer}\;}$), dividing by the FreeSurfer volume (Equation 1). Values close to 0 suggest minor volume differences, positive values indicate larger FastSurfer volumes relative to the FreeSurfer volume, and negative values indicate larger FreeSurfer volumes.

(1) $$\mathrm{RVD}=\frac{V_{\mathrm{FastSurfer}}-V_{\mathrm{FreeSurfer}}}{V_{\mathrm{FreeSurfer}}}\times 100\%$$

Furthermore, in order to evaluate potential systematic volume differences between corresponding ROIs in the right and left hemispheres, we calculated a hemispheric asymmetry index (HAI) (Kong et al. 2018; Sarica et al. 2018). It is defined as the difference between the left and right hemispheric volumes ($V_{ROI\_L}$ and $\;V_{ROI\_R}$), divided by their sum (Equation 2). Values close to 0 indicate symmetry between the hemispheres, positive values up to 100 suggest a larger left hemisphere, and negative values down to −100 indicate a larger right hemisphere.

(2) $$\mathrm{HAI}=\frac{V_{ROI_{-}L}-V_{ROI_{-}R}}{V_{ROI_{-}L}+V_{ROI_{-}R}}\times 100$$

To investigate whether an age effect contributes to the differences between the two tools, we examined the relationship between DSC or RVD and age using a linear model with the formula: metric ∼ age, where the metric represents either DSC or RVD. To account for multiple comparisons, we applied a Bonferroni adjustment to the *p*‐values. A negative slope indicates that the metric decreases with age—this suggests reduced segmentation overlap quality for DSC, or improved volume accuracy for RVD (since lower RVD is better). Conversely, a positive slope means the metric increases with age, potentially indicating better DSC or higher volume error (worse RVD), depending on the metric involved.

### 2.5 Representativeness of the Dataset

Prior to constructing the growth charts, we wanted to ensure that our cohort was representative of the considered age range. To this end, we performed a comparison with the results reported by Bethlehem et al. (2022), where 123,984 MRI datasets from subjects aged 115 days post‐conception to 100 years were analyzed. Specifically, we compared the volumes of GM, WM, subcortical GM, CSF, and mean cortical thickness. Post‐QC, all specified volumes and mean cortical thickness were plotted as scatter points for both sexes, alongside normative trajectories with 5%, 25%, 50%, 75%, and 95% centiles derived from (Bethlehem et al. 2022).

### 2.6 Growth Charts

After excluding the subjects with severe segmentation errors and ensuring the representativeness of the dataset, growth charts for relative volumes across the 15 anatomical regions were computed including the fifth, 50th, and 95th percentiles. The rolling percentiles were calculated using a window size of five and the resulting values fitted with the loess algorithm (Gijbels and Prosdocimi 2010) to generate the growth charts. ROIs of the right and left hemispheres were plotted as separate data points except for the brain stem, where each point in the plot represents one subject.

To reduce the impact of biases, such as interscanner variability or sex‐based differences in volume across ages (Guo et al. 2019), we normalized the volumes by dividing them by the eTIV (Kijonka et al. 2020).

To illustrate a potential clinical application of the growth charts, we use them as a comparator for the data of a 12‐year‐old patient, who suffered from a kernicterus as a neonate, a condition causing basal ganglia damage due to elevated levels of bilirubin.

## 3 Results

### 3.1 Final Dataset

In total, 58 (21:32:5 HBN:LOC:K2H) subjects were excluded after QC due to significant artifacts. Our final dataset for the validation of FastSurfer versus FreeSurfer comprised 448 subjects (149:93:206 HBN:LOC:K2H). The median age was 8.7 years (IQR 6.9‐11.2; 55% boys).

Following validation and before constructing the growth charts, 12 subjects were excluded because of severe segmentation errors. Examples of mis‐segmentations are shown in Table S2. Our final dataset for the growth charts comprised 436 Subjects. The median age was 8.7 years (IQR 6.9–11.2; 54% boys).

### 3.2 Validation

**FIGURE 2:**
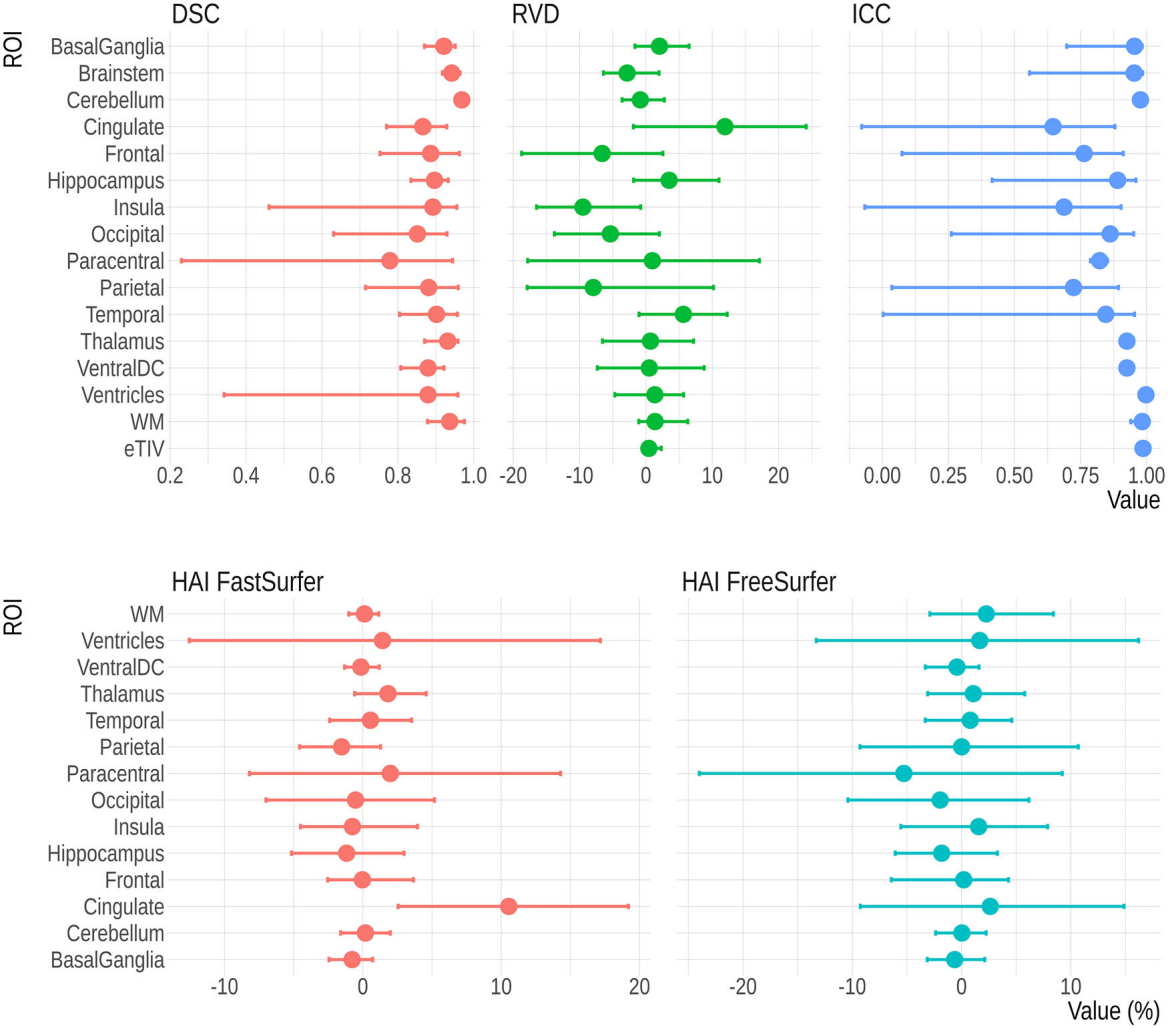
Metrics for the Validation of FastSurfer versus FreeSurfer: The dots represent the mean values across subjects (DSC, RVD) or metric values (ICC), while the error bars indicate the 95% bootstrap confidence intervals. The RVDs show average differences in volume across subjects.

No segmentation failed due to internal errors of FreeSurfer or FastSurfer. The duration of the volumetric analysis, comprising both segmentation and calculation of ROI volumes was about 3 min for FastSurfer and 3.5 h for FreeSurfer. DSCs for the 15 ROIs were generally very good (Figure 2 and Table S3). Slightly lower values were found for the paracentral lobule (0.79 95% CI:0.16; 0.94). The volume differences were low with RVDs (Figure 2) ranging between 11.9% (95% CI: 0.5%; 23.8%) for the cingulate, where FastSurfer derives larger volumes than FreeSurfer, and −9.5% (95% CI: −17.3%; 0.6%) for the insula, where FreeSurfer segmentations are larger. In addition, the RVD for the ventricles was relatively low (2.5%, 95% CI: −2.3%; 13.5%).

Testing for an association between DSC and age revealed a significant effect of age on the DSC of the cingulate, frontal lobe, insula, occipital lobe, parietal lobe, and temporal lobe. For all ROIs, the effects were negative and modest, corresponding to a decrease in DSC of 0.01–0.08 over the age range from 4 to 18 years old. Furthermore, a similar analysis on RVD showed a significant association for all ROIs, with the exception of the basal ganglia, brainstem, paracentral lobule, and ventral diencephalon. The effects were also modest and generally negative, corresponding to a decrease in RVD of 0.5%–8% over the age range from 4 to 18 years old. These findings are reported in Table 2.

**TABLE 2:** Effect of age on the validation metrics, calculated using a linear model with the formula: Metric ∼ age. padj: Bonferroni‐adjusted *p*‐value. ns: Not significant, *: Padj ≤ 0.05, **: Padj ≤ 0.01, ***: Padj ≤ 0.001, ** * *: Padj ≤ 0.0001. A **negative slope** means that as age increases, the segmentation metric (DSC or RVD) **decreases**—For DSC, this suggests reduced overlap quality with age, and for RVD, it indicates improved volume accuracy (since lower RVD is better). A **positive slope**, on the other hand, means the metric **increases** with age—This could signal worsening volume estimation for RVD (higher error) or improved overlap for DSC, though in this study, positive slopes were mostly seen in RVD and typically not significant.

ROI	DSC		RVD	
	Age (slope)	padj	Age (slope)	padj
BasalGanglia	−0.0015	ns	0.0002	ns
Brainstem	−0.0008	ns	−0.0006	ns
Cerebellum	−0.0001	ns	−0.0019	****
Cingulate	−0.0051	***	−0.0062	****
Frontal	−0.0037	*	−0.0038	****
Hippocampus	−0.0009	ns	−0.0036	****
Insula	−0.0048	***	−0.0038	****
Occipital	−0.0055	***	−0.0039	****
Paracentral	−0.0014	ns	0.0013	ns
Parietal	−0.0046	**	−0.0055	****
Temporal	−0.0038	**	−0.0035	****
Thalamus	−0.0012	ns	−0.0036	****
VentralDC	0.0000	ns	0.0006	ns
Ventricles	−0.0023	ns	0.0011	*
WM	−0.0025	ns	−0.0012	***

The agreement was good to excellent with ICC values exceeding 0.75 for most ROIs except for cingulate (0.65 95% CI: −0.08; 0.88), insula (0.69 95% CI: 0.07; 0.90), and the paracentral lobule (0.72 95% CI: 0.04; 0.89) (Figure 2).

Using the volumes from FreeSurfer, eight ROIs showed larger left‐hemisphere volumes (Figure 2 and Table S4). HAI values were mostly between −3 and 3, except for the paracentral lobule (−5.1, 95% CI: −9.62; −0.23), where the right hemisphere was larger. With FastSurfer, seven ROIs had larger left‐hemisphere volumes. HAI values range from −2 to 2. A remarkable asymmetry was found for the cingulate gyrus which was always considerably larger in the left hemisphere (10.54, 95% CI 2.10; 20.09) for FastSurfer. This behavior was not seen at a similar level in the volumes calculated using FreeSurfer. We compared the widths of 95% confidence intervals of HAI values between FreeSurfer and FastSurfer (Table S4). FastSurfer had narrower confidence intervals in all 14 ROIs.

**FIGURE 3:**
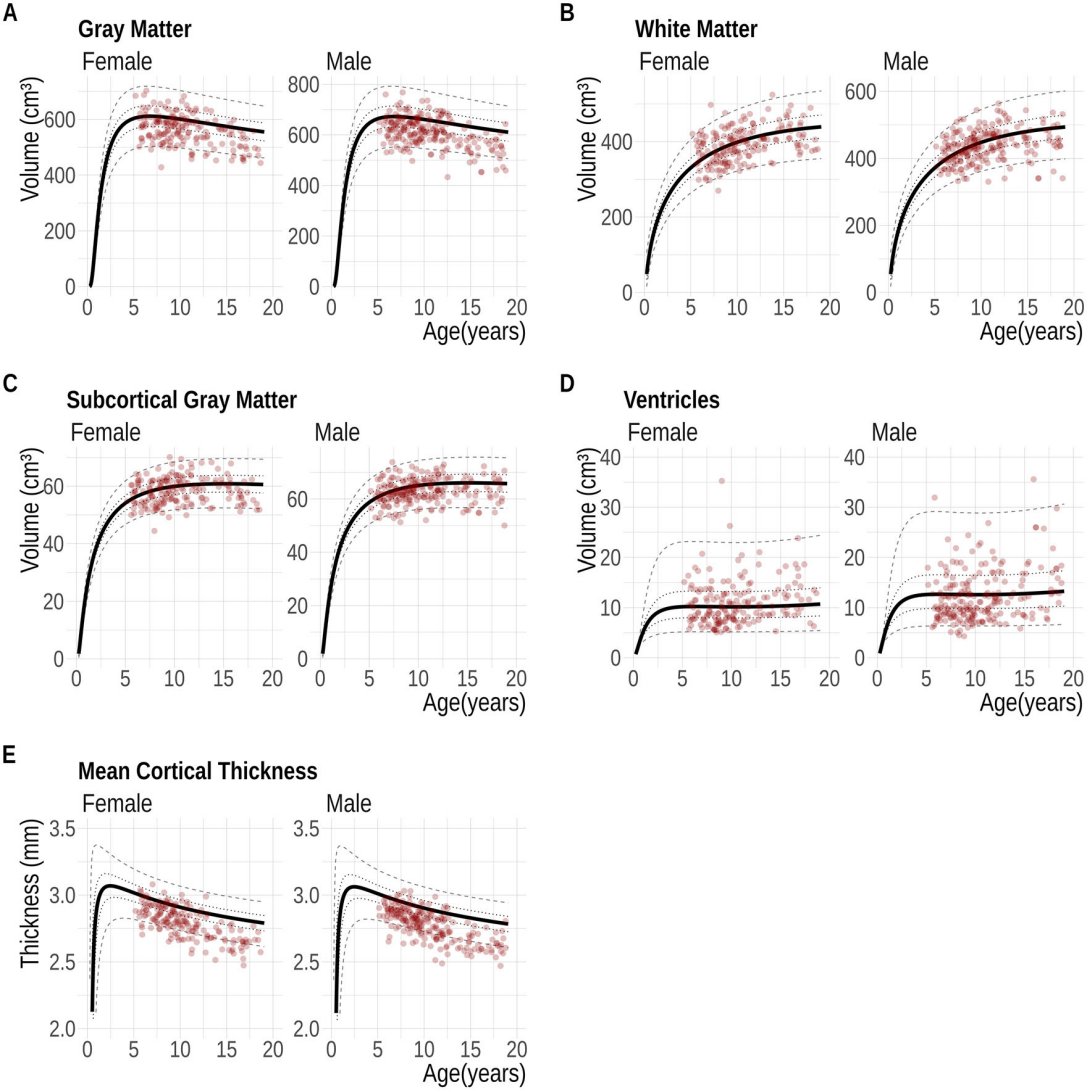
Representativeness of the dataset. This figure compares the FastSurfer estimates from our cohort (dots) with the broader dataset from Bethlehem et al. (2022). Solid lines represent the 50th percentile, while dotted lines indicate the fifth, 25th, 75th, and 95th percentiles, derived from Bethlehem et al. (2022).

The comparison of mean cortical thickness showed a moderate agreement between FastSurfer and FreeSurfer (ICC 0.62, 95% CI: −0.07; 0.87). Plotting the extracted thickness values against normative trajectories provided by (Bethlehem et al. 2022) showed a reduced mean cortical thickness (Figure 3E)

### 3.3 Representativeness of the Dataset

In Figure 3, absolute FastSurfer volumes from our dataset are compared to that in (Bethlehem et al. 2022). A visual inspection reveals that most data points follow a similar distribution across the entire age range. Moreover, the cortical thickness estimated (Figure 3E) by FastSurfer is mostly lower than that reported by (Bethlehem et al. 2022) across all ages.

### 3.4 Growth Charts

**FIGURE 4:**
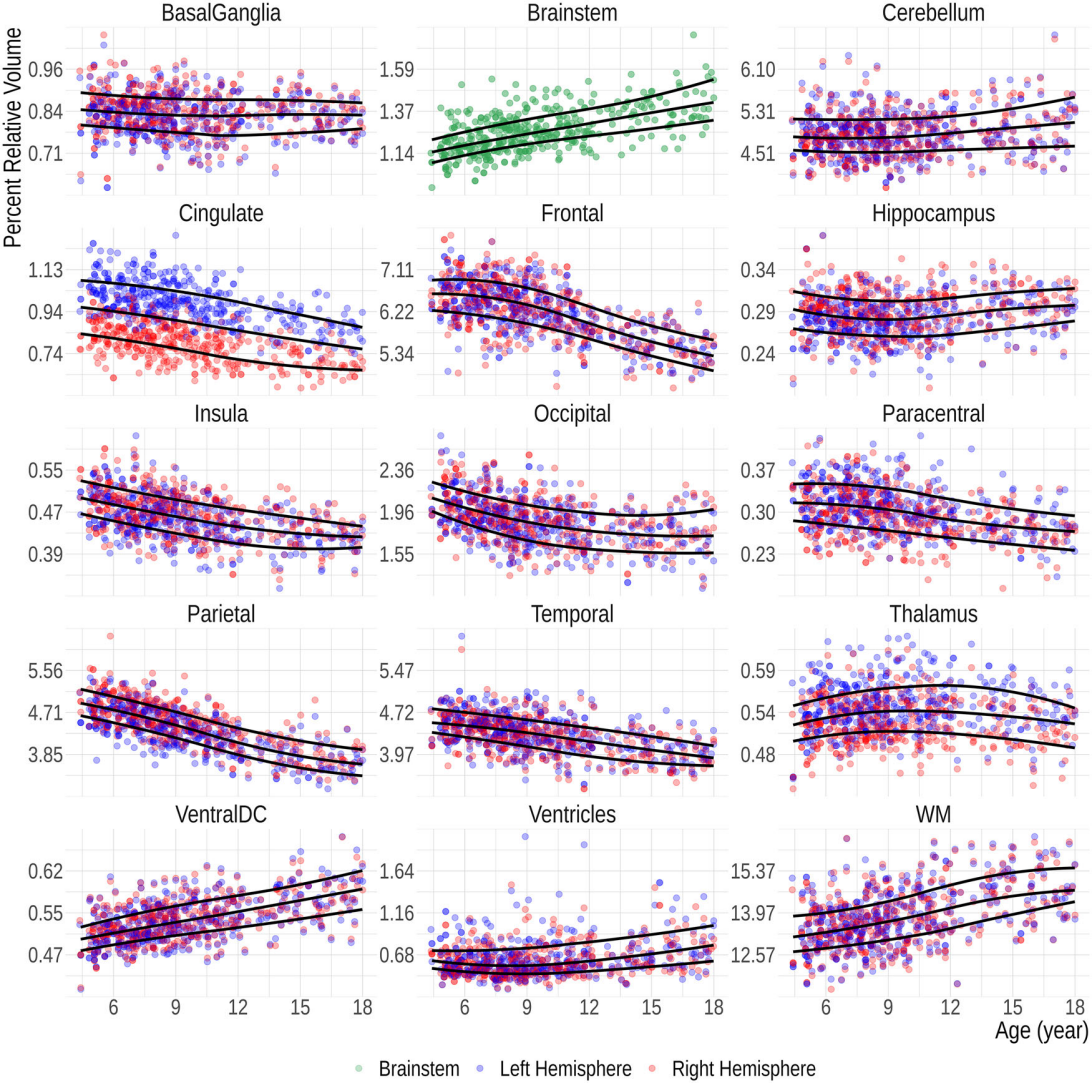
Growth charts for relative volumes of 15 regions of interest (ROIs) as a function of age. All ROI volumes are normalized with the estimated total intracranial volume. The solid lines represent the fifth, 50^th^, and 95^th^ percentiles. Except for brainstem, each datapoint represents the volume of an ipsilateral ROI.

The FastSurfer‐based age‐specific fifth, 50th, and 95th percentile curves for relative volumes of the 15 anatomical regions are shown in Figure 4. Except for the brainstem, each data point represents the relative volume of an ipsilateral ROI. Sex‐related differences are eliminated after performing the volume normalization (Figure S1), why both sexes are plotted together.

**FIGURE 5:**
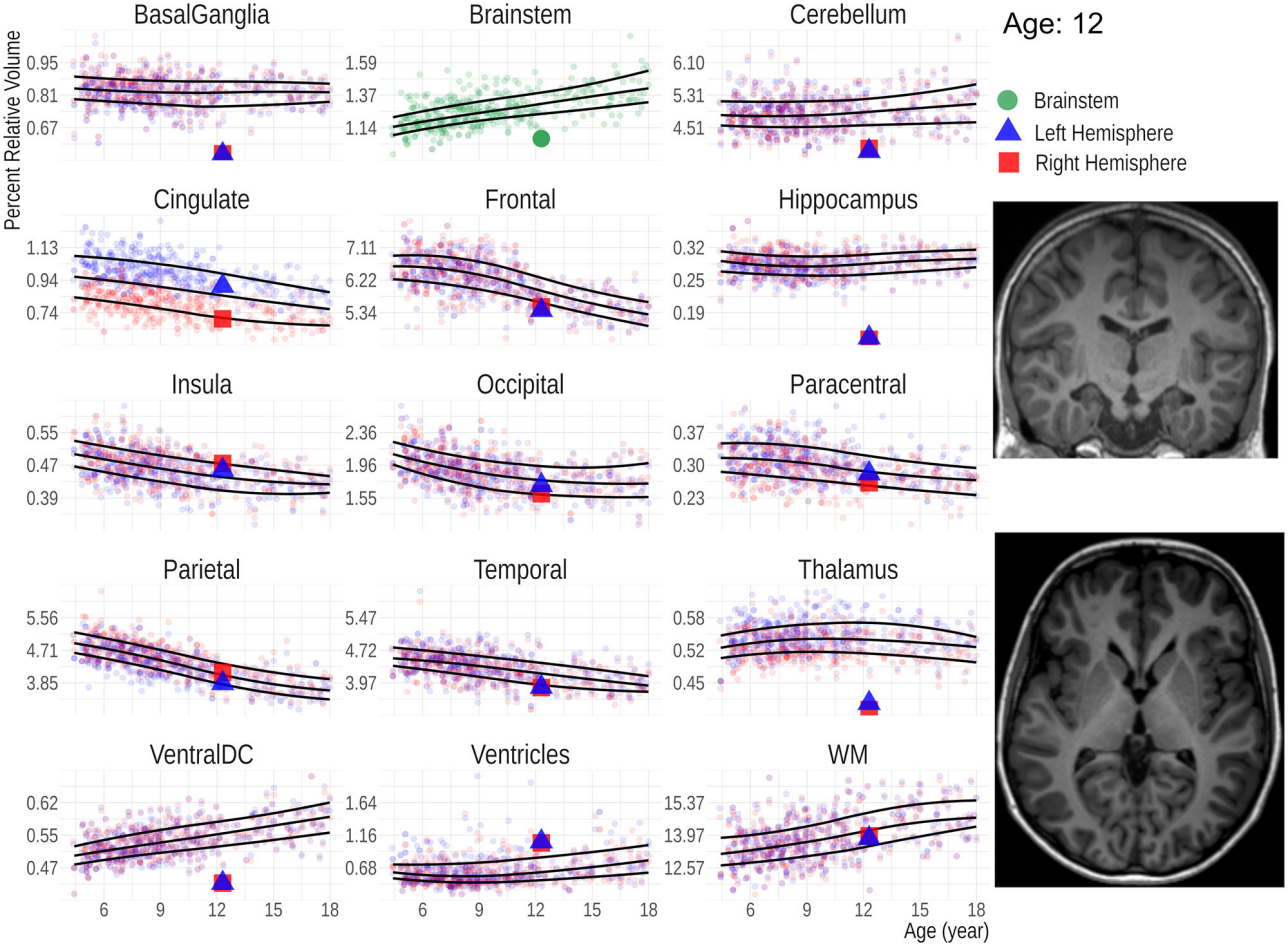
Clinical application of growth charts using a patient example. The relative brain region volumes of a 12‐year‐old patient, who suffered from kernicterus as a neonate, are plotted on the percentile curves. The basal ganglia, hippocampi and thalami are reduced in size, whereas the ventricles due to atrophy are larger than expected.

As a clinical use‐case of the derived results, we assessed the relative ROI volumes of a patient who suffered from kernicterus during the neonatal period using the growth charts (Figure 5). The basal ganglia and the hippocampi are considerably smaller than expected for age, while the ventricles are larger compared to those of age‐matched peers.

## 4 Discussion

The aim of our study was to compare FastSurfer and FreeSurfer in a pediatric cohort and to develop growth charts for 15 brain regions that can be used in the clinical context to assist in the diagnosis of pediatric brain conditions associated with hypoplasia and/or atrophy, as well as hyperplasia.

Before validation, 11% of the subjects were excluded due to artifacts on the un‐processed MR images to improve the reliability of the final segmentation results (Pulli et al. 2022). After the validation process, a thorough visual inspection of the FastSurfer output across 448 datasets focused on potential segmentation and labeling errors and led to the exclusion of only 12 (2.7%) subjects, which overall indicates a very high reliability of the FastSurfer segmentation results. Despite these encouraging results, we nevertheless consider a visual check to be an important intermediate step that should be included prior to further analysis.

The comparison of FastSurfer and FreeSurfer showed generally high values for all considered metrics and all ROIs. DSC values typically exceed 0.85 for most anatomical regions in our cohort, indicating a high overlap ratio of results from both tools. The only exception was the paracentral lobule, which had a slightly lower DSC of (0.79, 95% CI: 0.16; 0.94). While its anterior, posterior, and inferior boundaries are well‐defined by the precentral, postcentral, and cingulate sulcus, respectively, its superior extent remains less distinct, even for experts. This ambiguity may contribute to the lower DSC values and was confirmed by inspection of the segmentation masks. However, the clinical relevance of assessing the paracentral lobule separately may be limited, as it is typically considered part of the precentral and postcentral gyri. In this study, it was analyzed separately because it is part of the Desikan–Killiany atlas and could not be meaningfully integrated into the 15 defined brain regions. In addition, we observed mislabeling of the precentral and postcentral gyri by FreeSurfer in over 50 subjects, where the central sulcus was misplaced, indicating labeling inaccuracies in this region. While this contributed to lower DSC values for the paracentral lobule, its impact on the larger frontal and parietal lobes was less pronounced. Interestingly, FastSurfer demonstrated greater precision in correctly identifying sulcal boundaries.

Overall, our DSC values are comparable to those reported in the original FastSurfer publication by (Henschel et al. 2020). In that study, FastSurfer was validated on cortical and subcortical structures of adults using five different datasets, achieving a mean DSC of 0.89 and 0.86, respectively, as well as against manual segmentation with a mean DSC of 0.80 and 0.81. Detailed metrics for individual ROIs were not reported, which is why we are unable to make specific comparisons for anatomical subregions. FastSurfer was also validated against FreeSurfer for the segmentation of the hippocampus of Alzheimer's disease patients (Bloch and Friedrich 2021), where high correlations on the volume estimates were found. However, specific accuracy metrics such as the DSC were not reported.

We detected a slight age effect on both DSC and RVD across the age range. This may seem counterintuitive at first but can be explained by the modest magnitude of the effect and the lack of a strong relationship between the two metrics. In addition, this finding highlights that our tool, which is based on growth charts, is not designed to detect subtle differences.

Although the ICC values from the comparison are generally good or excellent (Figure 2), supratentorial cortical regions exhibit lower ICCs with wide confidence intervals. This is primarily due to updates in the FreeSurfer algorithm between versions 6 and 7. These updates, which affect the estimation of both the WM and pial surfaces (Haddad et al. 2023), have a greater impact on cortical thickness estimates but also influence cortical ROI volumes to some extent, leading to lower ICC values. Figure S2 in Supporting Information shows the correlation between FastSurfer and FreeSurfer volume estimates: While the overall correlation is strong, supratentorial cortical ROI data points are slightly shifted relative to the identity line, with either larger FreeSurfer or FastSurfer volumes. This shift contributes to the widening of the confidence intervals associated with the ICC.

Our data appears to be representative of children aged 4 to 18 years when compared to the large dataset by (Bethlehem et al. 2022) as all volume estimates fall within the interquartile range, except for mean cortical thickness, which is lower in our dataset. This pattern holds for both FastSurfer and FreeSurfer results across the entire age range (also in Figure S3). We speculate that this discrepancy may stem from limited between‐version reproducibility in FreeSurfer's cortical thickness estimates, a concern highlighted in a recent study (Haddad et al. 2023). (Bethlehem et al. 2022) used FreeSurfer v5.3 and v6 in their current models, as suggested by sample data (http://brainchart.io), whereas we used FreeSurfer v7.4 and FastSurfer, which was trained on FreeSurfer v6.0. This will inevitably introduce a bias. Although our sanity cross check was limited to four tissue classes, it gives a valuable clue as to whether the growth curves can be applied in a more generalized way (Figure S4).

The HAI in our dataset was close to 0 for most of the 14 ROIs, showing a high level of symmetry. However, a consistent and considerable exception was observed in the cingulate gyrus, where the left side was always larger in FastSurfer segmentations. Despite reevaluating several random subjects to identify the cause, we could not pinpoint the source of this systematic bias in FastSurfer outputs. FastSurfer demonstrated narrower confidence intervals across all 14 ROIs, potentially indicating higher consistency in its segmentation and volume calculation.

We also could not find specific differences between the FreeSurfer cingulate gyrus masks and the corresponding FreeSurfer v6 masks we segmented for a previous version of this study. FreeSurfer (v7) does not show the reported significant asymmetry as in the other two methods. However, it is important to highlight that the anatomical boundaries of the cingulate cortex are difficult to define and can pose challenges even for experienced observers. It is likely that the observed bias may stem from the anatomical template used during the preprocessing stage. Leftward asymmetry of cingulate subregions has been reported by others using FreeSurfer prior to version 7.4 (Kong et al. 2018) but has not been found as consistently as in our results. While functional aspects, e.g., handedness may certainly play an important role, the invariable greater volume on the left side in our cohort suggests technical causes, such as segmentation and parcellation issues, rather than functional ones. As FastSurfer is trained on FreeSurfer v6, it is not surprising that the same asymmetry is found.

Identification and quantification of cerebral atrophy is a critical aspect in clinical evaluations (Kurth et al. 2015), as it can distinguish specific pathological entities. For example, unilateral or asymmetric hippocampal sclerosis is a typical imaging correlate of temporal lobe epilepsy (Briellmann et al. 1998; N. Lee et al. 1995; Marsh et al. 1997). Also, Rasmussen encephalitis, a rare cause for drug‐resistant epilepsy is associated with unilateral atrophy (Bien et al. 2005). Our results suggest that except for the cingulate area, FastSurfer can be used to diagnose unilateral atrophy processes.

Although not the main goal of this study, we investigated sex‐related differences in brain volumes in our cohort. As reported by others (Blatter et al. 1995; Brain Development Cooperative 2012; Kijonka et al. 2020), male subjects had greater brain volumes and eTIVs than females. As in these previous studies, sex‐related differences in GM and WM volumes were, however, reduced in our cohort after normalization with eTIV (Buckner et al. 2004; Kijonka et al. 2020; Whitwell et al. 2001) highlighting the importance of head‐size adjustment if comparisons across sexes are made.

In our growth charts (Figure 4), three distinct growth patterns are evident—a decreasing volume with age observed in the cortical GM of the cingulate, frontal lobe, insula, parietal, and to some extent the occipital lobe; relative volume stability in the basal ganglia, thalamus, the GM of the paracentral lobule, hippocampus, and temporal lobe; and an increasing trend noted in WM structures, such as the brainstem and ventral diencephalon, in the WM as a whole, in the ventricles, and to a lesser extent, the cerebellum. The diverging growth patterns of the cortex and WM in the developing brain are well described (Brain Development Cooperative 2012; Lenroot et al. 2007) and are usually attributed to ongoing myelination with increasing axonal volume in WM and synaptic pruning in cortical regions (Huttenlocher 1979; Lenroot et al. 2007; Walhovd et al. 2017). This general trend is consistent with our growth charts. As described in the literature, the volume of the basal ganglia and thalamus are, however, essentially unchanged (Brain Development Cooperative 2012; Duerden et al. 2020). On the other hand, the previously described increasing hippocampal volumes are not apparent in our cohort (Duerden et al. 2020; Foland‐Ross et al. 2018; Obenaus et al. 2001). The observed divergent growth patterns of different tissue classes may also arise from technical reasons because of changing intensities in MRI during development (Filip et al. 2022; Haynes et al. 2020; Kuhne et al. 2021).

We illustrate the clinical application of brain volume growth charts with a case and believe that they could be particularly helpful for those less experienced in the MRI evaluation of pediatric brains by providing a reference point. Our example involves a 12‐year‐old patient who suffered from kernicterus as a newborn. In kernicterus, volume loss is typically observed in the globi pallidi, part of the basal ganglia, as well as in the subthalamic nuclei (Sugama et al. 2001). However, the comparison with our growth charts also shows potential abnormalities in the hippocampi and thalami in our patient that could be easily overlooked. In a previous study using Diffusion Kurtosis Imaging, altered diffusion metrics were found in the hippocampi and thalami of neonates (Zheng et al. 2021), suggesting that subsequent volume loss in these regions is likely. It is, however, important to note that subtle changes cannot be diagnosed, and individual data points must fall clearly outside the fifth or 95th percentile to be considered as deviations. In addition, volume quantifications should not be viewed as a substitute for a radiological evaluation, which relies on a comprehensive assessment of other equally important MRI features and clinical information.

Instead, our tool should solely be used as a supplementary aid after a QC of segmentation results. It is also imperative to mention that only substantial deviations from the fifth or 95th percentiles should be taken seriously, as technical‐ as well as population‐based issues can significantly impact the shape of the curves.

The relatively small cohort can certainly be seen as a limitation of our study. To date, normal data of children are not as widely available as those of adults. Moreover, we included data from three different MRI scanners, which inevitably introduces data variability. While collecting normative data using the same MRI system as clinical assessments is ideal, it is often impractical due to challenges in compiling large databases of healthy subjects on clinical scanners. Using data from different MRI scanners can, however, be regarded both as a limitation and a strength, as it helps generalize the growth charts to other settings. A limitation of our study is that the LOC subjects from our local MRI scanner are not entirely healthy; however, a thorough reevaluation of the imaging data and medical records definitely minimized this potential bias. The comparison with brain volume curves by (Bethlehem et al. 2022) suggests that the limitations fall within an acceptable range. In addition, we did not have insight into the subjects’ ethnicity which may influence the GM and WM volumes in different brain regions as reported by others (Choi et al. 2021; Huang et al. 2019). Relying on visual quality assessment introduces inherent arbitrariness, particularly in evaluating motion artifacts. Automated quality assessment algorithms could provide an objective estimate of motion severity (White et al. 2018) and could pave the way for establishing universal standards across distinct categories of motion severity (Pulli et al. 2022). However, challenges persist, such as the ambiguity in defining anatomical boundaries, notably with the frontal and parietal lobes.

Our results are based on FreeSurfer 7.4 and FastSurfer 2.3.3. Major updates to either tool may affect key aspects of segmentation in the future. For example, FreeSurfer 7 introduced significant changes in surface area calculation compared to version 6 (Haddad et al. 2023). Similarly, future updates to FastSurfer could impact our findings. Ongoing attention to software versions is essential.

In conclusion, we have shown that the segmentation and volume data of our cohort of children between 4 and 18 years obtained by FastSurfer are comparable to those of the widely used FreeSurfer. The short processing time of the deep learning‐based FreeSurfer software allows its application in the clinical context. Moreover, we developed a workflow to build MRI‐based growth charts for GM and WM volumes in children and adolescents between 4 and 18 years that might serve as a diagnostic aid tool to identify regional volumetric changes of brain tissue atrophy or hypoplasia.

## Author Contributions

**Ibrahim Zughayyar**: conceptualization, investigation, software, formal analysis, data curation, visualization, writing–review and editing, project administration. **Martin Bauer**: data collection, investigation. **Christopher Güttler**: visualization, formal analysis. **Ana Luisa de Almeida Marcelino**: writing–review and editing. **Fabienne Kühne**: project administration, writing–review and editing. **Claudia Buss**: writing–review and editing. **Christine Heim**: writing–review and editing. **Annette Aigner**: methodology, formal analysis, writing–review and editing. **Anna Tietze**: conceptualization, supervision, methodology, project administration, validation, writing–review and editing. **Andrea Dell'Orco**: conceptualization, supervision, methodology, software, data curation, formal analysis, visualization, writing–review and editing.

## Conflicts of Interest

The authors declare no conflicts of interest.

The code developed for this study is available under MIT License on GitHub (Zughayyar and Dell'Orco 2025) and includes the scripts, Jupyter Notebooks and a Docker environment.

## Peer Review

The peer review history for this article is available at https://publons.com/publon/10.1002/brb3.70689

## Supporting information




**Supplementary Information**: brb370689‐sup‐0001‐SuppMat.docx

## Data Availability

Due to data protection regularities, the MRI data of the LOC and K2H subcohorts are not openly available. The volumes and validation metrics for the entire cohort are available on OSF.io (Dell'Orco and Zughayyar 2025)
