# Supplementary material for: A FastSurfer Database for Age‐Specific Brain Volumes in Healthy Children: A Tool for Quantifying Localized and Global Brain Volume Alterations in Pediatric Patients
Source: Brain Behav. 2025 Jul 20;15(7):e70689. doi: 10.1002/brb3.70689 (PMC12277543; doi:10.1002/brb3.70689)
Supplement: Supplementary file 1 — Supplementary Information: brb370689‐sup‐0001‐SuppMat.docx [file BRB3-15-e70689-s001.docx]

# Supplementary Information

## A FastSurfer Database for Age-Specific Brain Volumes in Healthy Children: A Tool for Quantifying Localized and Global Brain Volume Alterations in Pediatric Patients

Table S1: Detailed subregions as defined by the Desikan-Killiany atlas (Desikan et al., 2006) are summarized into larger anatomical regions.

| **Summarized anatomical region** | **Subregions** |
| --- | --- |
| Basal ganglia | Caudate  Putamen  Pallidum  Accumbens area |
| Brainstem | Brainstem |
| Cerebellum | Cerebellum white matter  Cerebellum Cortex |
| Cingulate gyrus | Caudoanterior cingulate  Isthmus cingulate  Posterior cingulate  Rostralanterior cingulate |
| Frontal lobe | Caudalmiddlefrontal  Lateralorbitofrontal  Medialorbitalfrontal  Pars opercularis  Pars orbitalis  Pars triangularis  Precentral  Rostralmiddlefrontal  Superiorfrontal |
| Hippocampus | Hippocampus |
| Insula | Insula |
| Occipital lobe | Cuneus  Lateraloccipital  Lingual  Pericalcarine |
| Paracentral lobule | Paracentral |
| Parietal lobe | Inferiorparietal  Postcentral  Precuneus  Superiorparietal  Supramarginalis |
| Temporal lobe | Entorhinal  Fusiform  Inferiortemporal  Middletemporal  Parahippocampal  Superiortemporal  Transversetemporal  Amygdala |
| Thalamus | Thalamus-Proper |
| Ventral diencephalon | Ventral diencephalon |
| Ventricles | Lateral Ventricle  InfLatVentricle  ChoroidPlexus  3^rd^Ventricle  4^th^Ventricle  CSF |
| White matter | Cerebral White Matter, Corpus callosum |

Table S2: Examples of segmentation errors.

| Subject | FreeSurfer | FastSurfer | Description |
| --- | --- | --- | --- |
| sub-NDARTD290AY0 | 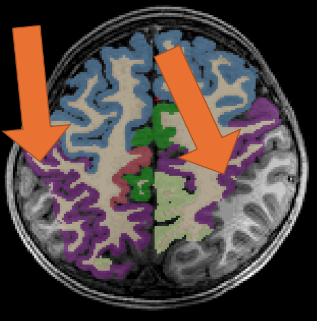 | 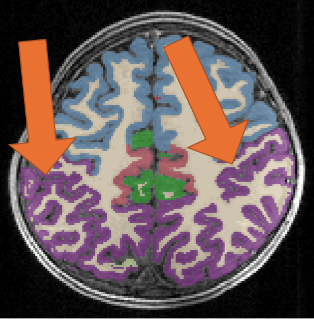 | The arrows highlight areas where FreeSurfer 7 massively undersegments several brain regions, showing incomplete or missing segmentation. |
| sub-NDARTD290AY0 | 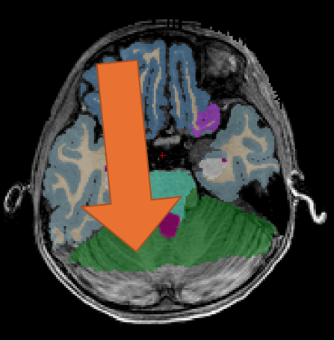 | 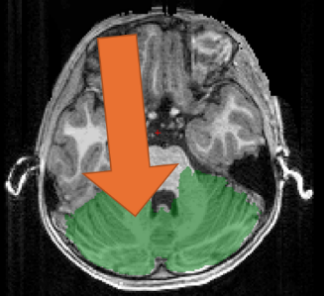 | The arrow indicates a region where segmentation FreeSurfer is incomplete or inaccurate, particularly around the cerebellum |
| sub-NDARLF446MT5 | 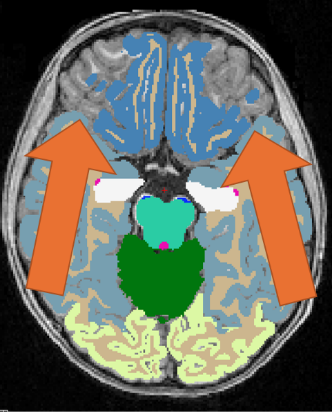 | 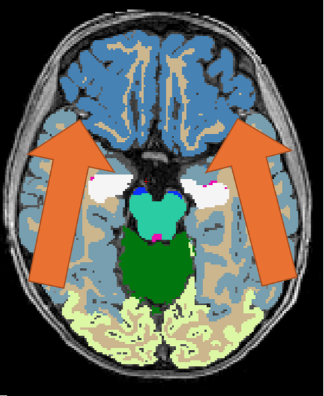 | FreeSurfer’s segmentation appears less distinct, with incomplete delineation of the green area, while FastSurfer shows clearer, more accurate boundaries, indicating better performance. |
| sub-NDARME930DE7 | 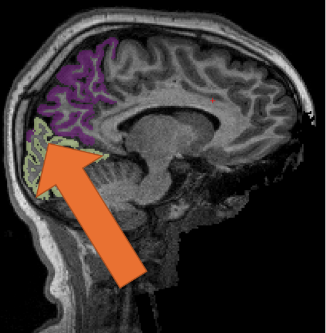 | 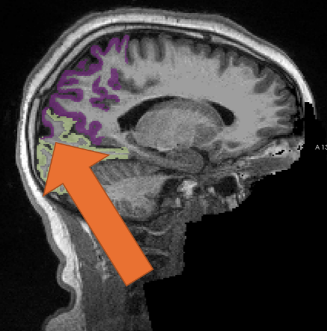 | The arrow highlighting mislabeling or inconsistency in the parieto-occipital sulcus. Purple areas (#4, Parietal) show FastSurfer’s improper extension into the occipital region, while FreeSurfer’s segmentation is slightly better but still inconsistent, |
| sub-NDARYW789GNP | 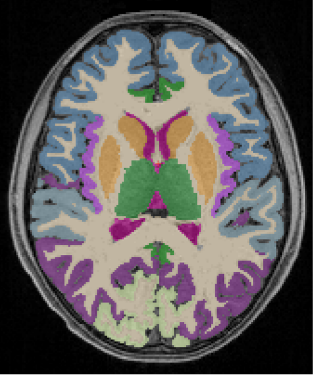 | 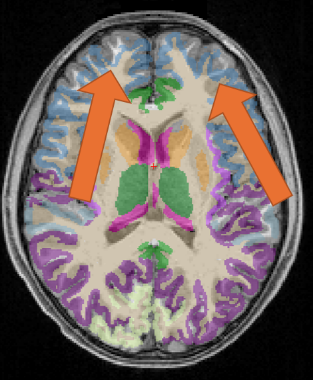 | FastSurfer’s segmentation shows inconsistency in the many brains regions |
| sub-NDARBN100LCD | 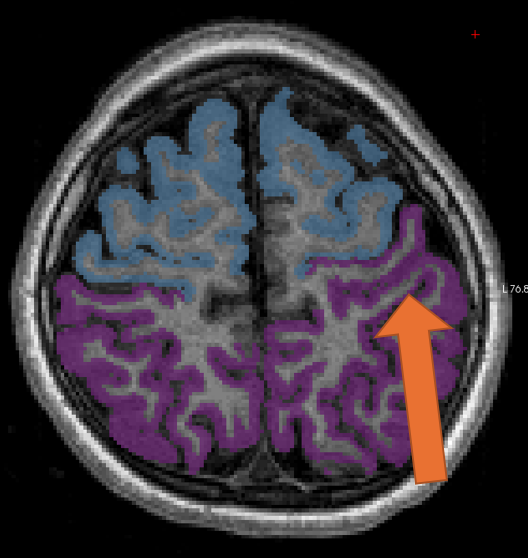 | 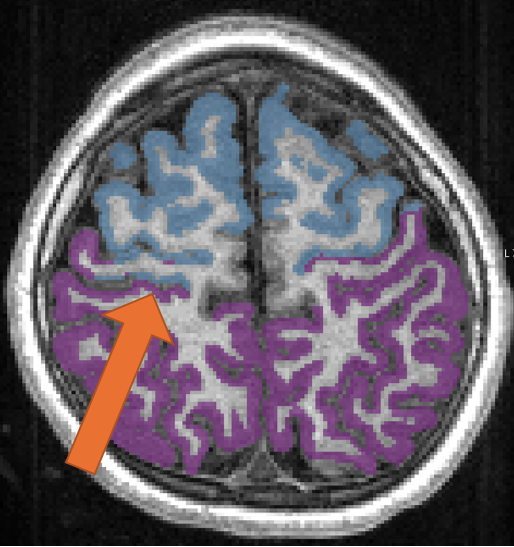 | Labeling errors around the central sulcus dividing the frontal and parietal lobes.  While FastSurfer recognized the Sulcus correctly it hast many misplaced voxels. |
| Sub-N209 | 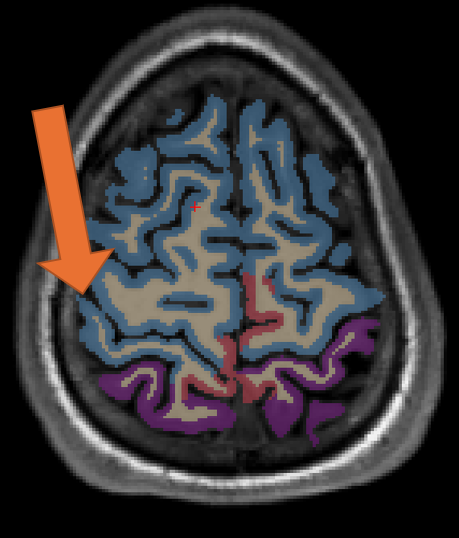 | 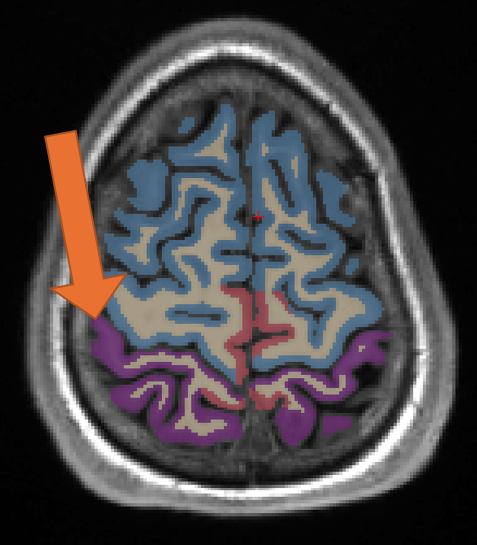 | While FreeSurfer incorrectly identifies the central sulcus, FastSurfer provides a more accurate segmentation |
| sub-NDARAM277WZT | 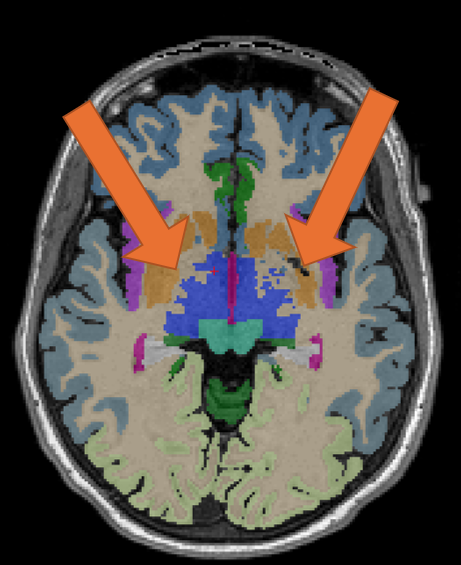 | 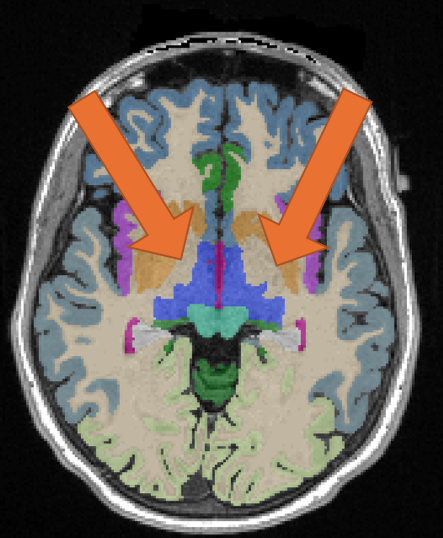 | FreeSurfer’s segmentation shows a poorly defined boundary. FastSurfer’ also struggles with this border, but the delineation appears slightly more precise, though still inconsistent, suggesting both methods have difficulty accurately defining VentralDC boundary |
| sub-NDARGW450YUX | 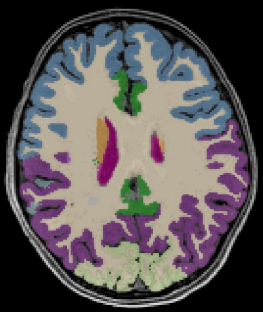 | 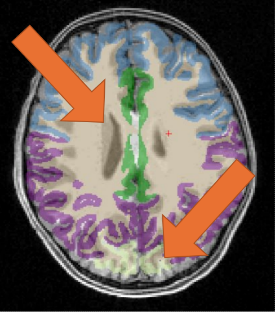 | FastSurfer includes many regions from the ventricles in the WM. Inconsistent in the occipetal region. |

Table S3 Summary of the validation metric using FastSurfers across 15 regions of interests. DSC: Dice similarity index, eTIV: estimated total intracranial volume, ICC: intraclass correlation coefficient, ROI: region of interest, RVD: percentage relative volume difference, CI: 95% bootstrap confidence interval (1000 resamples)

| ROI | Mean DSC (CI) | Mean RVD (CI) | ICC (CI) |
| --- | --- | --- | --- |
| eTIV |  | 0.44% (0.03%; 2.53%) | 0.99 (0.98; 0.99) |
| Basal ganglia | 0.93 (0.87; 0.95) | 2.05% (-1.66%; 6.18%) | 0.95 (0.70; 0.98) |
| Brainstem | 0.95 (0.92; 0.96) | -2.85% (-7.47%; 1.81%) | 0.95 (0.55 - 0.98) |
| Cerebellum | 0.97 (0.96; 0.98) | -0.85% (-3.54%; 2.76%) | 0.98 (0.96 - 0.98) |
| Cingulate | 0.88 (0.77; 0.93) | 11.90% (0.47%; 23.84%) | 0.65 (-0.08 - 0.88) |
| Frontal lobe | 0.89 (0.76; 0.96) | -6.63% (-19.97%; 2.71%) | 0.76 (0.07 - 0.91) |
| Hippocampus | 0.91 (0.83; 0.93) | 3.49% (-1.78%; 9.26%) | 0.89 (0.41 - 0.96) |
| Insula | 0.90 (0.80; 0.96) | -9.51% (-17.33%; -0.58%) | 0.69 (-0.07 - 0.90) |
| Occipital lobe | 0.86 (0.75; 0.93) | -5.37% (-13.42%; 2.20%) | 0.86 (0.26 - 0.95) |
| Paracentral lobule | 0.79 (0.16; 0.94) | 0.99% (-17.83%; 16.71%) | 0.82 (0.79 - 0.85) |
| Parietal lobe | 0.89 (0.78; 0.96) | -7.90% (-18.14%; 9.66%) | 0.72 (0.04 - 0.89) |
| Temporal lobe | 0.91 (0.83; 0.96) | 5.63% (-0.98%; 12.25%) | 0.85 (0.00 - 0.95) |
| Thalamus | 0.94 (0.91; 0.96) | 0.68% (-7.01%; 7.29%) | 0.93 (0.91 - 0.94) |
| VentralDC | 0.89 (0.81; 0.92) | 0.52% (-7.54%; 9.03%) | 0.92 (0.91 - 0.94) |
| Ventricles | 0.89 (0.81; 0.95) | 1.35% (-4.74%; 5.86%) | 1.00 (0.99 - 1.00) |
| White matter | 0.94 (0.88; 0.98) | 1.37% (-1.38%; 6.92%) | 0.98 (0.94 - 0.99) |

Table S4: Hemispheric Asymmetry Indices (HAI) across the 14 regions of interests (ROIs) with both methods. CI: 95% bootstrap confidence interval (1000 resamples)

| ROI | HAI | | | |
| --- | --- | --- | --- | --- |
|  | FastSurfer | | FreeSurfer | |
|  | Mean (95% CI) | CI Width | Mean (95% CI) | CI Width |
| Basal ganglia | -0.78 (-2.36; 0.67) | 3.03 | -0.63 (-2.90; 1.78) | 4.68 |
| Cerebellum | 0.18 (-1.49; 2.02) | 3.51 | 0.03 (-2.41; 2.16) | 4.57 |
| Cingulate gyrus | 10.54 (2.10; 20.09) | 17.99 | 2.62 (-9.36; 13.57) | 22.93 |
| Frontal lobe | -0.03 (-2.51; 3.65) | 6.16 | 0.20 (-6.43; 4.99) | 11.42 |
| Hippocampus | -1.17 (-5.07; 3.00) | 8.07 | -1.82 (-6.46; 2.81) | 9.27 |
| Insula | -0.76 (-4.34; 3.80) | 8.14 | 1.56 (-5.08; 8.23) | 13.31 |
| Occipital lobe | -0.53 (-6.04; 5.17) | 11.21 | -1.96 (-10.42; 5.63) | 16.05 |
| Paracentral lobule | 1.98 (-8.19; 13.61) | 21.80 | -5.28 (-22.26; 7.99) | 30.25 |
| Parietal lobe | -1.54 (-4.71; 1.20) | 5.91 | 0.01 (-8.65; 10.65) | 19.30 |
| Temporal lobe | 0.54 (-2.17; 3.36) | 5.53 | 0.79 (-3.54; 4.51) | 8.05 |
| Thalamus | 1.81 (-0.71; 4.43) | 5.14 | 1.07 (-2.59; 5.52) | 8.11 |
| VentralDC | -0.14 (-1.27; 1.21) | 2.48 | -0.42 (-3.02; 1.78) | 4.80 |
| Ventricles | 1.42 (-11.30; 16.55) | 27.85 | 1.67 (-10.92; 18.48) | 29.40 |
| White matter | 0.11 (-1.12; 1.22) | 2.34 | 2.26 (-3.37; 7.73) | 11.10 |


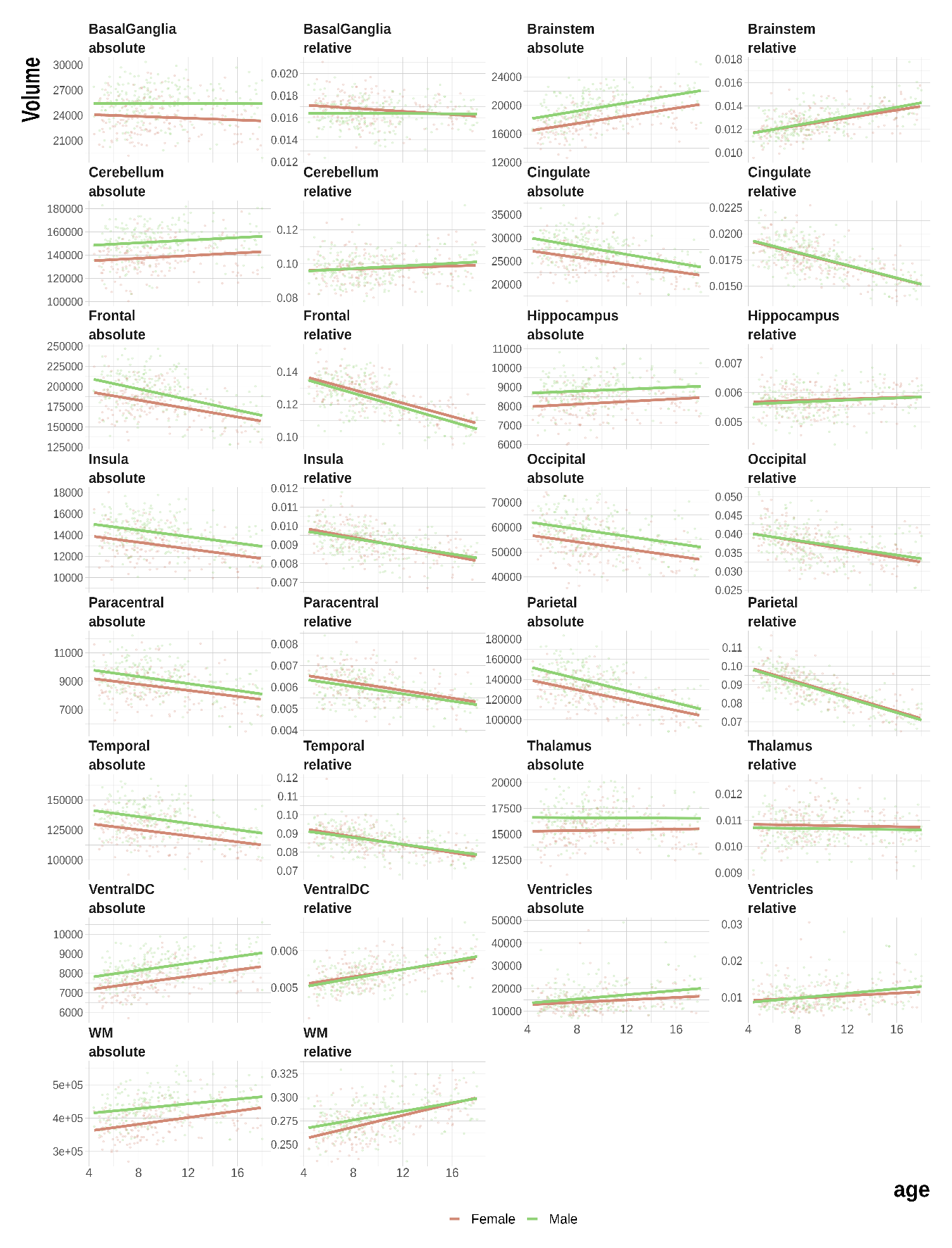


*Figure S1: Comparison of absolute and relative volume across the 16 regions of interest (ROI) considering the subjects’ sex). Absolute ROI volumes of males tend to be larger than those of girls, but this difference vanishes when volumes are relative to the estimated total intracranial volume instead.*


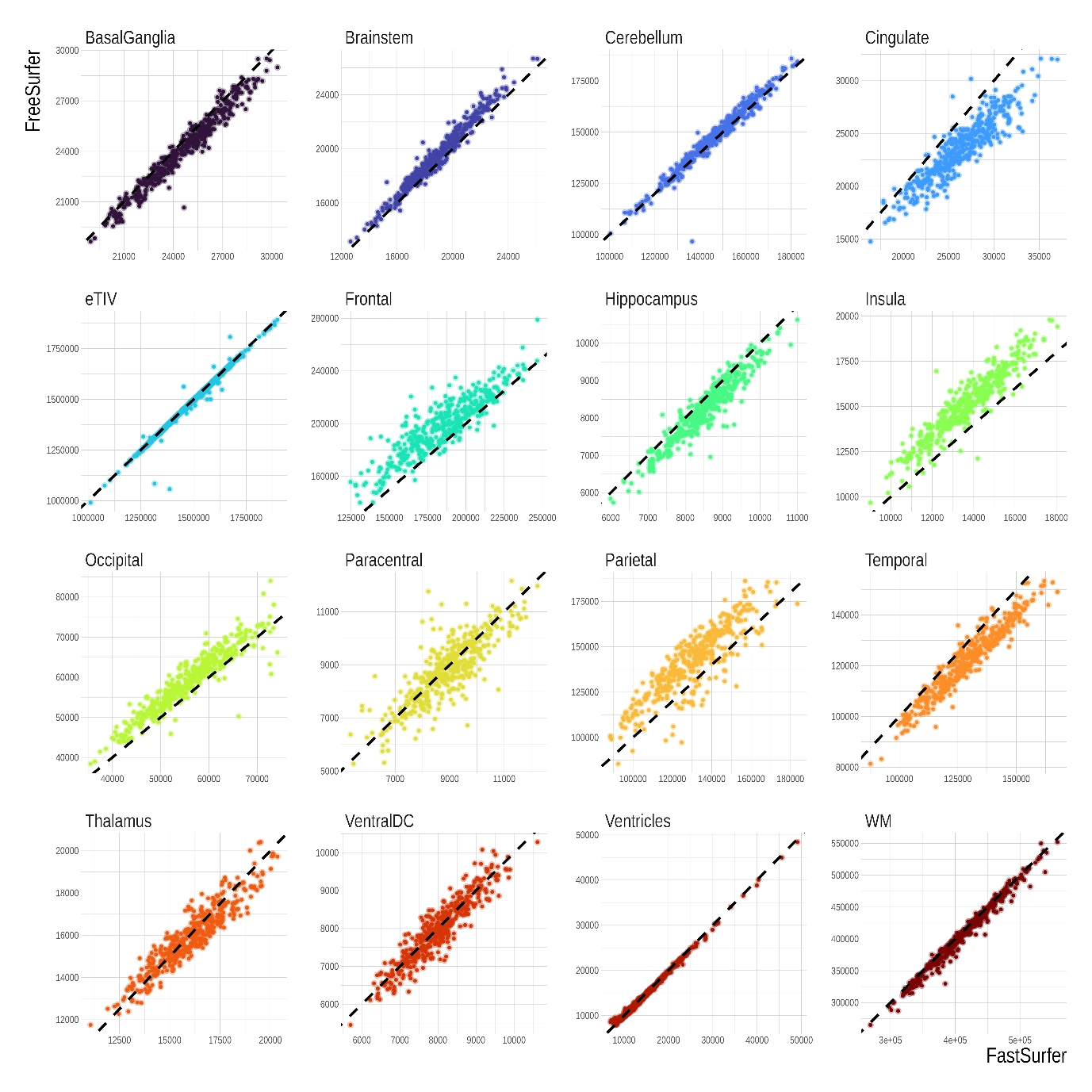


Figure S2: Scatterplots representing the correlation between FastSurfer and FreeSurfer volumes. The dashed gray line represents the identity line, while the dashed black line represents the association between the volumes of the two methods. It can be seen that the ROIs with the most extensive distribution of dots around the black line have worse values for all the metrics considered in Figure 2.


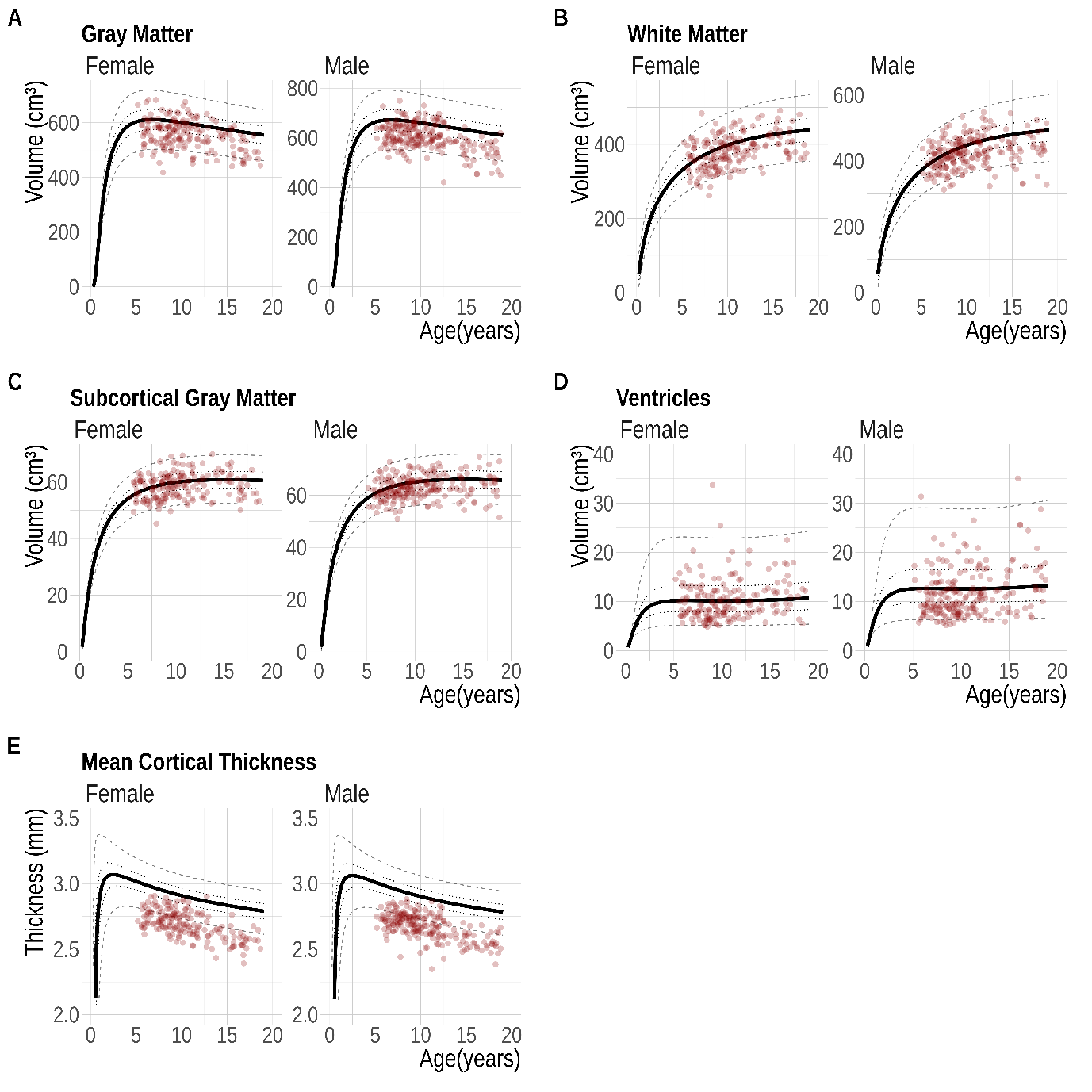


Figure S3: Comparison of our dataset to that in (Bethlehem et al., 2022): Each point represents absolute volumes of a single subject in our dataset (using Freesurfer v7) in the grey matter volume (A), white matter volume (B), subcortical grey matter volume (C), and ventricular volume (D). Solid lines represent 50^th^percentiles, dotted lines the 5^th^, the 25^th^, the 75th and 95^th^ percentiles, derived from (Bethlehem et al., 2022).


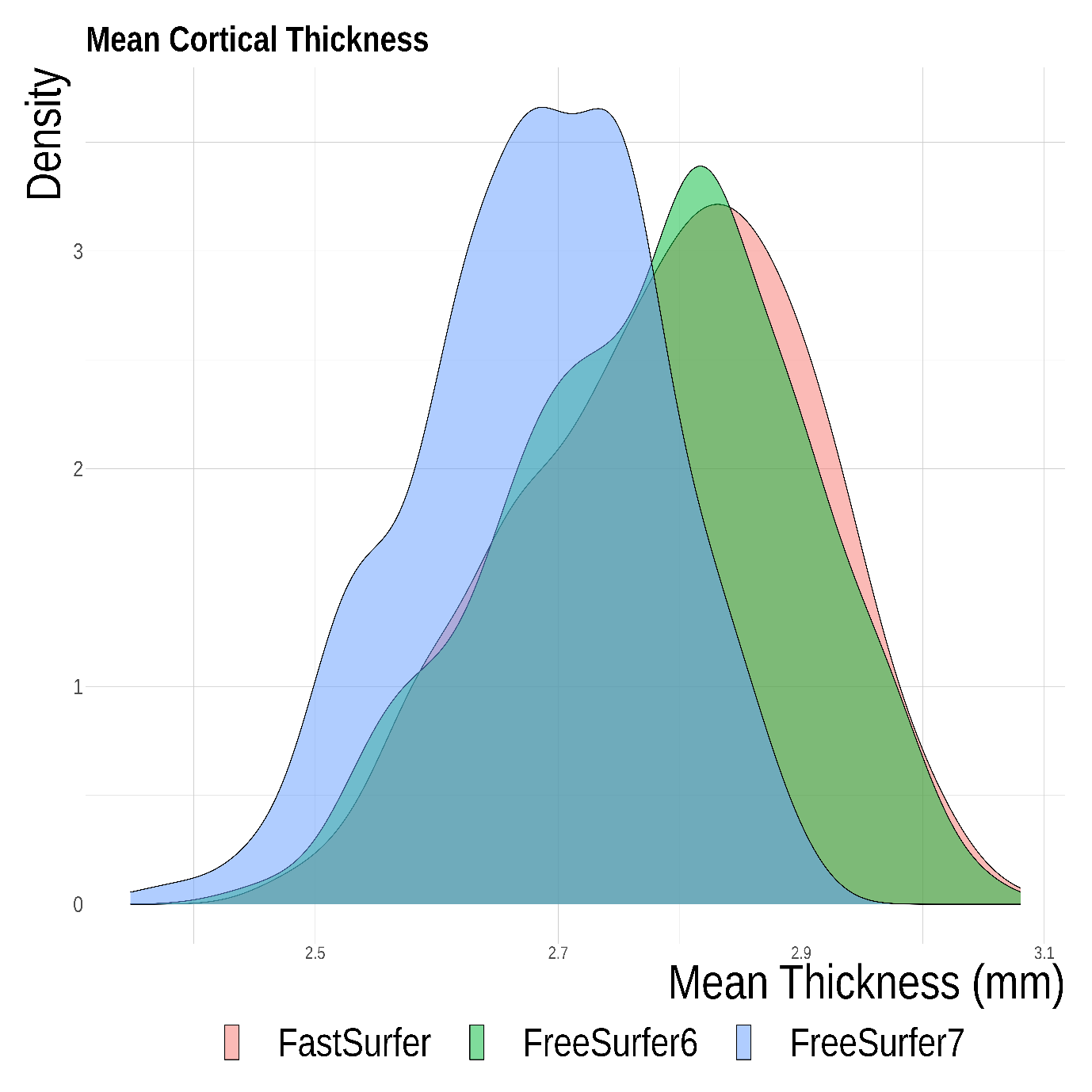


Figure S4: illustrates the distribution of mean cortical thickness measured by FreeSurfer 7.4, FreeSurfer 6, and FastSurfer v2.3.3, presented as density plots. The graph shows that FreeSurfer 7 consistently reports lower mean cortical thickness values
